# Patients’ cancer care perceptions conceptualized through the Cancer Experience Measurement Framework

**DOI:** 10.1186/s12913-022-07946-y

**Published:** 2022-05-23

**Authors:** Michaela A. Bourque, Carmen G. Loiselle

**Affiliations:** 1 Department of Psychiatry, McGill University, Montreal, Canada; 2 Department of Psychology, Crandall University, Moncton, Canada; 3 Department of Oncology, McGill University, Montreal, Canada; 4 Ingram School of Nursing, McGill University, Montreal, Canada; 5 Segal Cancer Centre, Jewish General Hospital, Centre Intégré Universitaire de Santé Et de Services Sociaux (CIUSSS), 680 Sherbrooke, Centre-Ouest, Montreal, QC Canada

**Keywords:** Patient experiences, Patient satisfaction, Cancer care, Content analysis, Person-centered care, Patient experience measurement framework

## Abstract

**Background:**

Research on patients’ perceptions of cancer care often documents sub-optimal experiences. Cancer care quality issues include restricted service access, lack of care coordination, gaps in follow-up and “generic” rather than person-centered care. Recent reports underscore that proactively and periodically seeking user feedback is crucial for timely care quality improvement. The present study aimed to analyze and thematically organize a large amount of feedback from patients who had been treated for cancer within the last 6 months.

**Methods:**

Randomly selected participants (*N* = 3,278) from 3 University-affiliated cancer centres in Montreal, Quebec, Canada completed the Ambulatory Oncology Patient Satisfaction Survey (AOPSS) and an open-ended question on their perceptions of the care they received. 692 participants responded to the latter. Guided by the Cancer Experience Measurement Framework (CEMF), their feedback was analyzed using a qualitative thematic approach.

**Results:**

Cancer care perceptions included sub-themes of care access and coordination, continuity/transition, and perceived appropriateness/personalisation of care.

The most salient theme was captured by care access and coordination with 284 comments (44%) directly addressing these issues. The ways in which health care services were structured including setting, schedule, and location were often raised as cause for concerns. Issues surrounding cancer information/education, emotional support, and physical comfort were frequently reported as unmet needs. In addition, limited access to cancer services led patients to seek alternatives such as going to emergency departments and/or private care.

**Conclusions:**

These findings are timely as they show that most patients are well aware of quality issues in cancer care and are willing to report candidly on these. Patient feedback also underscore the importance for cancer care institutions to periodically gather patient-reported data so that systems can re-calibrate their service offerings according to these data. Ultimately, patient reports will translate into enhanced quality, personalization, and safer cancer care provision.

## Background

**Fig. 1:**
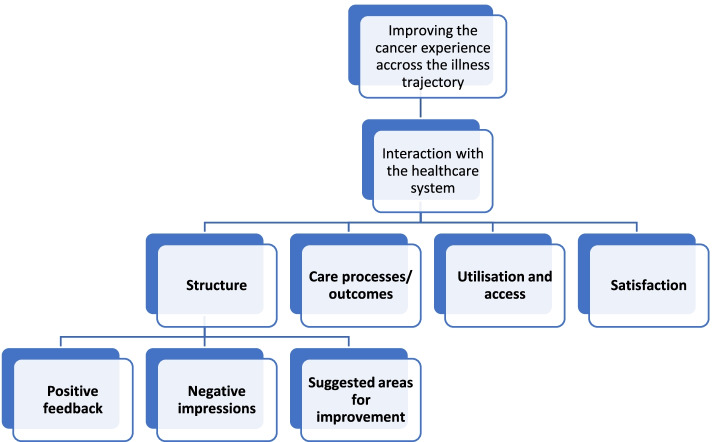
Patient experiences described by the four CEMF domains through 3 main classifications and 12 sub-categories

**Fig. 2:**
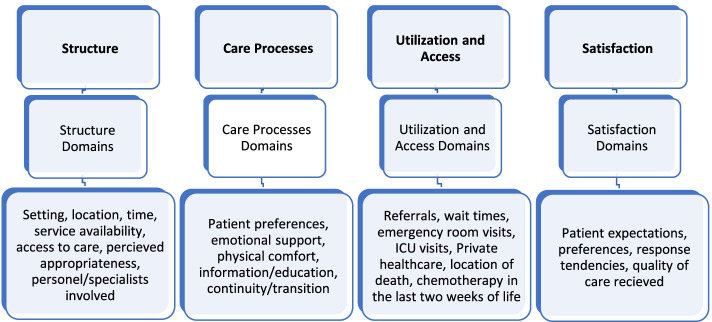
The four CEMF domains and their comprehensive facets (i.e. relevant themes) [18]

The cancer trajectory presents numerous patient challenges both personally and at the system level [18]. The cancer experience is dynamic, involving various interactions, events, and transitions across its course. Cancer experiences can be optimized through situation-responsive and personalized person-centred care (PCC) approaches [16]. Serving as a primary determinant of overall quality of care, PCC is defined as respectful, active, and tailored approaches meeting patients’ needs, values, and ideals [10, 18] (Figs. 1 and 2).

Wong and colleagues [26] noted direct feedback from users (e.g., patients) was best for assessing patients’ interactions with healthcare. Patient-focused, self-report questionnaires capture the good and bad of healthcare systems—highly valuable when assessing cancer care services, patient outcomes, and refining care practices. Standardized patient experience questionnaires provide a comprehensive understanding of users’ experiences within and across respondents [9, 26].

Research suggests multidimensional challenges related to patient healthcare experiences [3, 21]. Lee et al. [13] noted the primary goal of care coordination is to optimize outcomes through timely health service delivery (e.g., better access, more relevant interventions, and higher patient satisfaction). However, domains of care to be assessed are often a priori dictated by “generic” patient surveys (top-down) rather than emerging from feedback provided by patients themselves (bottom-up).

### Mapping out people’s experiences with cancer: the Cancer Experience Measurement Framework

The Cancer Experience Measurement Framework (CEMF; [18]) stems from an extensive review of current literature on the experiential context of cancer-affected individuals. This framework proposes four categories that must be examined if seeking a comprehensive account of cancer-related experiences: the patient perspective, the family perspective, the shared patient-family perspective and users’ interactions with the healthcare system [18]. Interactions with healthcare (the focus of this paper) encompass four domains: 1) care structure, 2) care processes and outcomes, 3) service utilisation/access and 4) patient satisfaction. Stemming from the informational aspects of these CEMF domains [16, 18], this study explores patient experiences of cancer care using data from the Ambulatory Oncology Patient Satisfaction Survey (AOPSS). More specifically, patient open-ended feedback is examined and categorized into the 4 domains of the “interaction with healthcare” CEMF framework category.

## Methods

### Sample and setting

The sample consisted of patients being aged 18 years or older, having a cancer diagnosis and being treated within the last six months in one of the participating cancer centres in Montreal, Quebec. These included the Segal Cancer Centre at the Jewish General Hospital (CIUSSS Centre-Ouest), the Cedars Cancer Centre at the McGill University Health Centre, and the Cancer Centre at St. Mary’s Hospital (CIUSSS Ouest) (see also [17]). Out of 7,885 surveys mailed to eligible participants over a four-year period (2015–2019), 3278 returned a completed survey. Nearly 60% of respondents were female and 51% reported being over 65 [22]. Various cancers were represented, the most common being breast cancer (29%) and hematology/lymphoma (14%). 156 participants did not report a diagnosis. Respondents are distributed as follows: Segal Cancer Centre (34%), Cedars Cancer Centre (37%) and the Cancer Centre at St. Mary’s General Hospital (29%). Of the total respondents, 692 provided comments in the open-ended portion of the AOPSS.

### Design and measures

A self-report survey was used to collect participant demographics and experiences. The Ambulatory Oncology Patient Satisfaction Survey (AOPSS), is a standardized, valid, and reliable self-report measure used in healthcare research (NRC Picker, [7, 20]). The AOPSS includes 83 items pertaining to patient experience and satisfaction with 45 core questions addressing six care domains (i.e., emotional support, coordination and continuity of care, respect for patient preferences, physical comfort, information/education, and access to care) and an open-ended question, “Is there anything else you would like to tell us about your experience with cancer care services?”.

### Procedures

Patient health records were cross-referenced with hospital mailing lists to create a random selection of potential participants (RCN, https://www.mcgill.ca/search/Aopss?search_origin=rcr-rcn). New eligible participants were mailed a survey package every three months, including a cover letter, AOPSS, and a prepaid return envelope. Participants were informed that completion and return of the survey indicated voluntary consent. A follow-up package was sent if a completed survey was not received four weeks after mailing. No personally identifiable information was collected. The Rossy Cancer Network facilitated survey administration and distribution with AOPSS license holder NRC Picker. This work was performed as an operational quality improvement activity. According to the policy constituting research at McGill University and its affiliated health care centres, it was deemed exempt from ethics review.

### Analysis

Responses (*N* = 692) on the open-ended AOPSS question “Is there anything else you would like to tell us about your experience with cancer care services?” were analyzed using thematic deductive analysis [23]. We used the four domains of the “interaction with healthcare" CEMF facet as pre-determined codes. To begin the analysis, survey feedback was reviewed by the two authors (MB., CL.) to obtain an understanding of the whole. The responses were then coded by each author separately after which both parties then discussed the data and compared the coding. A structured categorization was adapted, modelled after the four domains of the CEMF category of “interaction with healthcare”. Subcategories were discussed and agreed upon by both authors, and the data were then grouped based on how they were linked to the agreed-upon subcategories. All research group discussions pertaining to the data analysis continued until a shared understanding was reached.

## Results

Of 692 responses received, 45 were excluded due to their neutrality, leaving 647 responses for analysis. Deductive coding revealed 93.5% of participant feedback contained one or more components tied to at least one domain of the CEMF.Positive (*n* = 352, 54%), negative (*n* = 145, 23%), and suggestions for improvement (*n* = 145, 23%) responses provided nuanced accounts of cancer care services received. Organizing data in accordance with the four CEMF domains, 12 subcategories were identified and subsequently sorted into three principal classifications (i.e., positive feedback, negative impressions, and areas for improvement).

### Interactions with healthcare systems—access and coordination of care

Access to and coordination of care are CEMF themes within the four principal domains of structure, care processes, utilization and access, and patient satisfaction [18]. Of 647 respondents, 284 (44%) mentioned access or coordination of care issues, with 105 (37%) considered positive, 65 (23%) negative, and 114 (40%) indicating areas for improvement. Examples of verbatim feedback follow,these were chosen due to their representative nature within their given domains.

#### Domain 1: structure of the health care system

This domain represents material, human, and organizational resources affecting health systems’ ability to provide individualized/personalized care [18]. Participant responses fitting this category often referred to domain subcategories of setting, time, location, service access and availability, perceived appropriateness, and personnel or specialists involved [18].

### Negative impressions

This classification encompasses participants who described sub-desirable impressions of their care experiences.

*“I had weekly chemo treatments (IV) & it was necessary to have a blood test the day before the treatment. I understand it had to be done, but this meant 2 visits weekly to [the cancer centre] & sometimes it was inconvenient, time-consuming -- also paying for parking twice weekly!*” (Comment 59; Relevant Themes: time, setting, perceived appropriateness, availability of care)

### Positive feedback

Feedback in this classification is representative of pragmatic participant views outlining cancer care.

*“I am very pleased with the cancer care I am receiving. The doctor and clinical team are doing an excellent job. I am already surviving for 10 years. The wait times are always acceptable, very rarely too long. The team and oncologist are very professional. I fully understand the treatment I am receiving. The pharmacist is also always very accessible and helpful. I am very grateful for my treatments. Thanks so much.”* (Comment 286; Relevant Themes: time, perceived appropriateness, personnel involved, availability of care)

### Areas for improvement

Participant responses highlighted under this classification outline areas for improvement in cancer care as per service users.

*“[An] excellent cancer care team at [the cancer centre] (receptionists, secretaries, technologists, doctors, etc). Very compassionate and professional. Extremely competent. An area that can be improved [is] the ability to reach receptionists/secretaries to schedule appointments or get updates. Typically, I was diverted to voicemails, and sometimes my calls were not returned.”* (Comment 662; Relevant Themes: time, perceived appropriateness, personnel involved, availability of care)

#### Domain 2: processes of care

Care processes represent the interwoven series of events contributing to individualized patient care experiences like timelines related to diagnosis, planning and implementation of treatment regimens [18]. Care outcomes can be described as cancer morbidity, adverse events, cancer-related hospital (re) admissions, and emergency room visits [18]. Commonly reported domain-consistent sub-themes included patient preferences, emotional support, physical comfort, information/education, continuity/transition, coordination of care, and access to care [18].

### Negative impressions

*“Very trying experience…Had to wait very long for diagnosis…Oncologist very professional but gave rehearsed answers, did not understand or care about my feelings of fear and apprehension. [I] felt very much like a number. For emergencies unable to contact anyone after 4 pm or on the weekends. [As a result, I] had to wait over 17 hours in [the] emergency room!!! - Absolutely no info about alternative therapies!”* (Comment 72; Relevant Themes: patient preferences, information/education, continuity/transition, coordination of care, access to care, emotional support)

### Positive feedback

*“I think the cancer wards/rooms are exceptional. I think the staff is kind & patient. In particular, the staff at the main reception counters. There are also volunteers to answer any questions we may have. My oncologist & pivot nurse are also exceptional - I am positive I am in the right place for treatment. No one wants or deserves this illness but having health care that [is] such great support certainly facilitates the journey…”* (Comment 381; Relevant Themes: patient preference, physical comfort, emotional support, information/education, access to care, coordination of care)

### Areas for improvement

*“The service was very good. The staff at radiation oncology were very gentle, compassionate, punctual, respectful* + *polite. They explained very well if I had questions. Unfortunately, the secretaries forget to do appointments and then when you are there, they shuffle the charts, [so] you think there is one or two before you and suddenly there are 4 or 5 [other patients] before you.”* (Comment 607; Relevant Themes: emotional support, physical comfort, patient preference, information/education, access to care, coordination of care)

#### Domain 3: health care utilization and access

This domain represents the accessibility of healthcare services and facilities, including access to public/private services, hospital equipment (for treatment or further tests), wait times, access to emergency rooms, intensive care unit visits, and relevant referrals [18]. This specific domain led to few comments,these tended to be negative or mixed in nature.

### Negative impressions

*“Translated from French – [Called the] emergency number in oncology – does not return our calls. I had to go to the hospital emergency room.”* (Comment 112; Relevant Themes: access to emergency services)

*“… [I] Had to wait very long for a diagnosis. Went to a private breast clinic and spent $3000 for diagnosis and biopsy and still had to wait five weeks to see [a] surgeon after a positive diagnosis with Stage III cancer…”* (Comment 72; Relevant Themes: wait times, accessing private/public services, continuity/transition of care)

### Areas for improvement

*“Translated from French - Improve coordination between chemotherapy and radiation therapy. Ensure that appointments are well registered in the computer system and eliminate appointment errors The support staff is exceptionally courteous and respectful. The medical staff is very busy and as a result not very available…”* (Comment 637; Relevant Themes: relevant refferals, access to care, coordination of care)

#### Domain 4: patient satisfaction with cancer care

This domain encompasses patients’ and families’ expectations, preferences, and perceptions of quality of care, including appropriateness, coordination, access, and continuity/transition of care, followed by emotional support and physical comfort [18]. Satisfaction is subjective,these comments address users’ reported satisfaction featuring domain consistent sub-themes including setting, wait times, information/education.

### Higher cancer care satisfaction

*“Translated from French - I am very satisfied and grateful for the services received. I would recommend your institution to everyone. I appreciated the discussions about taking part in research and possibilities of experimental treatments offered in another university-affiliated cancer centre. I had a great relationship with my 3 treating doctors for 5 years. I thank you greatly for the quality of services offered. During my 2 hospitalizations, the food was excellent.”* (Comment 410; Relevant Themes: patient preference, perceived appropriateness, information/education)

### Lower cancer care satisfaction

*“The wait time for oncologist appointments is unacceptable I've had apt @ 11:30 am and oncologist saw me @ 4:00 pm. Average wait in the past 3 years is 3 hours…Not sure why [appointment] time is given if not respected.”* (Comment 127; Relevant Themes: wait times, perceived appropriateness)

### Areas for improvement

*“When a patient is diagnosed with cancer for the first time, the doctors don’t explain enough of the situation… they should explain more about cancer because for us it is something new and for us, it is not a daily thing that we know… I hope the system could change so [the patient] could get more information when you are diagnosed with cancer for the first time.”* (Comment 79; Relevant Themes: information/education, patient preference)

*“Overall, I am pleased with the care I received and continue to receive. I think there are definite gaps that need to be addressed - in particular, concerning information sharing with the newly diagnosed patient* + *their families. Also, the doctors need to be more open about complementary* + *alternative medicine…Nutritionists also need to be trained to consider what is particularly useful to fight cancer. The food I received while in hospital should be banned from cancer wards/patients…”* (Comment 582; Relevant Themes: patient preference, information/education)

## Discussion

Consistent with the literature [1–3, 6, 8, 16–18], this analysis captured the diversity in content, valence, and perceptions that contributes to patient experiences with cancer care systems. Guided by the CEMF, this analysis has provided structure to better understand contributors to cancer care processes and outcomes. The following elaborates on domain-specific observations and areas for improvement consistent with participant feedback herin.

### Domain 1 & 3: health care system structure, utilization, and access

Consistent with Gomez-Cano et al. [8], participants’ feedback regarding healthcare system structure and utilization-focused on wait times and continuity of care. Bridge and colleagues [3] clarify that dissatisfaction with wait-time could be further delinated as either waiting to see a healthcare provider, waiting for tests/results, and waiting for treatment/pharmacy follow-ups. Of additional concern were time-sensitive cancer-related unmet needs (e.g., no phone calls returned after hours from the cancer centre, long wait times in emergency rooms). Furthermore, wait times led some to contact private services for more expedient cancer care modalities. Some participants also reported related wait times could impact avoidable adverse outcomes. This concern should be further addressed clinically and through research.

### Domain 2: processes of care

Cancer care processes rely on practitioner expertise and individual preference to develop and implement treatments and timelines related to cancer care and prognoses [18]. As in the literature, our findings reported discrepancies in this domain surrounding general patient preferences, information/education, access, coordination, and continuity of care. For example, Li and colleagues [14] noted that participants reported discrepancies between expected and provided cancer information when receiving cancer-related education. For optimal outcomes, cancer information must be tailored to meet patients’ personal, cultural, and health needs [19]. Some participants reported dissatisfaction with the amount of cancer-related information provided, while others did not. This discrepancy may be influenced by information/communication preferences, as outlined in Loiselle et al. [18]. Hack and colleagues [12] suggest patients’ preferences may also influence this discrepancy for decision-making involvement.

Participants who reported dissatisfaction with continuity of care transitions felt less emotionally supported throughout their care experience. Like in Lee (2010), participants noted coordination issues were especially prevalent when cancer care is shared by multiple subspecialties involving multiple providers and locations. Patients suggested providers be better equipped to streamline intra-team information on how treatment and care modalities evolve. Similar to Bridge and Colleagues [3] and Grunfeld & Earle [11], participants felt responsibility should not rest solely on patients to update health care providers about their condition.

Overall, patient preferences were the most variable satisfaction-related domain of cancer care, with preferences such as times, dates and frequencies of appointments impacting overall satisfaction. Loiselle and Brown [16], Gillespie et al. [4], and Tzelepis et al. [25] remind us that there is no average patient, only individuals whose preferences for care are – well—personal.

### Domain 4: patient satisfaction with cancer care

Participants reported positive regard for their overall care experience. Indeed, Tremblay and colleagues [24] found patients who received care from a high-intensity interdisciplinary team were more likely to report positive experiences in areas like quality of patient-professional communication, continuity of care, and person-centred response. Of interest, positive feedback from this study was most common when referencing interdisciplinary work, with many acknowledging being thankful for the healthcare providers and all they had done in terms of physical and emotional support throughout their cancer trajectory. In addition, participants were likely to refer to doctors, nurses, and care coordinators as positive figures in their cancer care.

### Limitations

Several limitations surround the current study. Because this work was completed as a quality improvement activity, there is an increased chance these results may not represent the patient experience in other contexts. Reliance on the CEMF to organize patient feedback may have limited the scope or depth of analysis; an alternate framework may have yielded different interpretations and findings. However, as the CEMF is comprehensive in its nature, we do not feel that the use of this framework is a limitation. Self-selection biases may have been present; thus, these findings may not universally indicate the patient experience. Some individuals may have been more compelled than others to respond to the survey, resulting in findings that may not encapsulate the macro patient experience. Additionally, recall bias may have been operating due to the retrospective nature of patient experiences [3], and participant feedback may not hold complete accuracy. Generalizability is limited as some sociodemographic data were not recorded (e.g., sex and language spoken at home).

These limitations can be addressed. First, patient experience research should be extended beyond one health network, allowing a more macro perspective. Additionally, applying multiple frameworks may provide a more holistic yet critical data analysis. Self-selection and recall biases are difficult to avoid; however, they may be minimized by recruiting participants actively receiving cancer care. We recognize these limitations may impact our findings; however, research of this nature must rely on patient reports to capture a clear image of the patient experience; thus, we felt it acceptable to proceed with the current work due to its perceived value.

### Implications for future research

These findings highlight the importance of considering patients’ feedback when developing, implementing and updating cancer care practices. Authentic person-centred and personalized care approaches must periodically query patients about their experiences, preferences, and unmet needs. Our own work is a step in that direction [15, 16, 18], and future research should continue to gather patient reports, including unaddressed symptoms, urgent care issues, unexplained wait times, and care (dis)continuity. As the Covid-19 pandemic has put undue stress on patients through dramatic institutional shifts towards infection control [5], issues of physical distancing, waiting room closing, and decreased capacity in cancer treatment areas may continue to significantly impact people’s cancer experiences and outcomes.

## Conclusion

Interactions in cancer care have significant ramifications for both overall care experiences and health-related outcomes [18]. Understanding what cancer care users, rather than institutions, may view as most relevant and timely is vital [3, 16, 18]. A thematic analysis related to care structure, processes, utilization, and satisfaction has led to a comprehensive account of important patient experiences in cancer. Key reported challenges must be addressed such as reducing wait times and ensuring smooth coordination of care as well as access to intra- and inter-organization cancer care services across the illness trajectory.

## Data Availability

Data are available upon request to Michaela Bourque by email Michaela.bourque@mail.mcgill.ca.

## Abbreviations

PCC: Person-centred care
AOPSS: Ambulatory Oncology Patient Satisfaction Survey
CEMF: Cancer Experience Measurement Framework

## References

[1] Balogh EP, Ganz PA, Murphy SB, Nass SJ, Ferrell BR, Stovall E (2011). Patient-centered cancer treatment planning: improving the quality of oncology care. Summary of an institute of medicine workshop. The Oncologist.

[2] Bracher M, D. J., & Wagland, R.  (2016). Exploring experiences of cancer care in Wales: a thematic analysis of free-text responses to the 2013 Wales cancer patient experience survey (wcpes). BMJ Open.

[3] Bridge E, Conn LG, Dhanju S, Singh S, Moody L (2019). The patient experience of ambulatory cancer treatment: a descriptive study. Curr Oncol.

[4] Gillespie H, Kelly M, Gormley G, King N, Gilliland D, Dornan T (2018). How can tomorrow’s doctors be more caring?. A phenomenological investigation. Med Educ..

[5] Coalition Priorité Cancer Quebec. (2020). The impact of the measures implemented to counter the covid-19 pandemic on oncology patients. https://coalitioncancer.com/wp-content/uploads/2020/06/FINAL_REPORT_COVID-CANCER-JUNE2020.pdf

[6] Corner J, Wagland R, Glaser A, Richards SM (2013). Qualitative analysis of patients’ feedback from a proms survey of cancer patients in England. BMJ Open.

[7] Ferguson D. Validation of the NRC Picker Canada Ambulatory Oncology Patient Satisfaction Survey. Ontario: NRC Picker Canada; 2012. [Google Scholar]

[8] Gomez-Cano M, Lyratzopoulos G, Abel GA (2020). Patient experience drivers of overall satisfaction with care in cancer patients: evidence from responders to the English cancer patient experience survey. J Patient Exp.

[9] Gomez-Cano M, Lyratzopoulos G, Campbell JL, Elliott M, Abel G (2022). The underlying structure of the English cancer patient experience survey: factor analysis to support survey reporting and design. Cancer Med.

[10] Grover C, Mackasey E, Cook E, Nurse H, Tremblay L, Clinician N, Loiselle CG (2018). Patient-reported care domains that enhance the experience of “being known” in an ambulatory cancer care centre. Can Oncol Nurs J.

[11] Grunfeld E, Earle CC. The interface between primary and oncology specialty care: treatment through survivorship. J Natl Cancer Inst Monogr. 2010;2010(40):25–30. 10.1093/jncimonographs/lgq002. 10.1093/jncimonographs/lgq002 PMC3482947 20386051

[12] Hack TF, Degner LF, Watson P, Sinha L (2006). Do patients benefit from participating in medical decision making? Longitudinal follow-up of women with breast cancer. Psychooncology.

[13] Lee T, Ko I, Lee I, Kim E, Shin M, Roh S, Yoon D, Choi S, Chang H (2011). Effects of nurse navigators on health outcomes of cancer patients. Cancer Nurs.

[14] Li C-C, Matthews AK, Dossaji M, Fullam F (2017). The relationship of patient-provider communication on quality of life among African-American and White cancer survivors. J Health Commun.

[15] Loiselle CG (2019). Cancer information-seeking preferences are associated with distinct patient experiences and satisfaction with cancer care. Patient Educ Couns.

[16] Loiselle CG, Brown TL (2020). Increasing access to psychosocial oncology services means becoming more person-centered and situation-responsive. Support Care Cancer.

[17] Loiselle CG, Attieh S, Cook E, Tardif L, Allard M, Rousseau C, Thomas D, Saha-Chaudhuri P, Talbot D (2020). The nurse pivot-navigator associated with more positive cancer care experiences and higher patient satisfaction. Can Oncol Nurs J.

[18] Loiselle CG, Howell D, Nicoll I, Fitch M (2019). Toward the development of a comprehensive cancer experience measurement framework. Support Care Cancer.

[19] Lyson HC, Haggstrom D, Bentz M, Obeng-Gyasi S, Dixit N, Sarkar U. Communicating critical information to cancer survivors: an assessment of survivorship care plans in use in diverse healthcare settings. Journal of Cancer Education: The Official Journal of the American Association for Cancer Education. 2021;36(5):981–9. 10.1007/s13187-020-01725-1. 10.1007/s13187-020-01725-1 PMC7483188 32128714

[20] National Research Corporation. Development and Validation of the Picker Ambulatory Oncology Survey Instrument in Canada. National Research Corporation; 2003.

[21] Pelzang R (2010). Time to learn: Understanding patient-centred care. British J Nurs.

[22] Rossy Cancer Network. The experience of patients with cancer at diagnosis and during treatment. A report of RCN survey results from 2014-2018. Montreal (QC): Rossy Cancer Network; 2018. p. 24. https://www.mcgill.ca/rcr-rcn/files/rcr-rcn/rcn_patient_experience_report_2018.09.pdf.

[23] Scharp KM, Sanders ML (2019). What is a theme? teaching thematic analysis in qualitative communication research methods. Commun Teach.

[24] Tremblay D, Roberge D, Touati N, Maunsell E, Berbiche D (2017). Effects of interdisciplinary teamwork on patient-reported experience of cancer care. BMC Health Serv Res.

[25] Tzelepis F, Sanson FRW, Hall AE, Carey ML, Paul CL, Clinton MT (2015). The quality of patient-centred care: Haematological cancer survivors’ perceptions. Psychooncology.

[26] Wong ELY, Coulter A, Hewitson P, Cheung AWL, Yam CHK, Lui S, fai, Tam, W. W. S., & Yeoh, E.  (2015). Patient experience and satisfaction with inpatient service: development of short form survey instrument measuring the core aspect of inpatient experience. PLoS ONE.

